# Brief Psychiatric Screening Questionnaire in Parkinson's Disease

**DOI:** 10.1155/2019/8143868

**Published:** 2019-07-24

**Authors:** Will Lee, David R. Williams, Andrew Evans

**Affiliations:** ^1^Neuroscience Department, The Alfred Hospital, Commercial Road, Melbourne, Victoria 3004, Australia; ^2^Neurology Department, The Royal Melbourne Hospital, Grattan Street, Parkville, Victoria 3050, Australia

## Abstract

**Background:**

While numerous validated questionnaires measuring psychiatric symptoms in Parkinson's disease (PD) are available, a quick multifaceted screening tool is lacking.

**Objective:**

To generate the Brief Psychiatric Questionnaire (BPQ) that quickly screens for depression, anxiety, and apathy and to evaluate its content validity against three reference scales.

**Methods:**

Forty-seven questions were drafted and measured against the Geriatric Depression Scale (GDS), State Trait Anxiety Inventory (Form Y2) (STAI-Y2), and Apathy Evaluation Scale (AES). Data were reduced by principal component analysis and linear regression. Content validity and repeatability were assessed in a second cohort.

**Results:**

Data from ninety-five patients were used for BPQ development. Variation explained by the final linear regression models was 52% for GDS (*R*^2^ = 0.521, *F*(2,94) = 49.97, *p* < 0.0001), 65% for STAI-Y2 (*R*^2^ = 0.652, *F*(4,94) = 42.08, *p* < 0.0001), and 14% for AES (*R*^2^ = 0.135, *F*(1,94) = 14.51, *p* < 0.0001). From the initial pool, only five questions remained for further testing. BPQ questions correctly identified 88% in the second cohort of 33 patients scoring more than five on GDS and 91% who scored in the highest decile of STAI-Y2, but only 51% who scored in the highest decile of AES. Moderate to strong correlation (*r* = 0.464 − 0.733, *p* < 0.004) between predicted scores based on BPQ questions and actual scores of three validated questionnaires was demonstrated. Good repeatability of BPQ questions was demonstrated by moderate to high intraclass correlation coefficients (0.47–0.772, *p* < 0.01).

**Conclusions:**

BPQ questions were able to accurately identify patients at risk of depression and anxiety but not apathy. It is brief and multifaceted and can act as a preconsultation tool to prompt further psychiatric assessment.

## 1. Introduction

Psychiatric symptoms are extremely common in Parkinson's disease (PD) even early on in the disease [1] and contribute significantly to patient's disability and caregiver's burden. Apathy, depression, and anxiety are particularly common and are present in over 40% of patients with PD [2]. Therefore, the ability to identify patients at risk of developing these psychiatric problems is invaluable as this can facilitate early intervention.

There are many validated questionnaires that examine various neuropsychiatric facets of PD. However, they are often lengthy to complete and are specific to one domain. Psychiatric symptoms in PD often overlap, for example, depression coexists with anxiety in 9% [2] and with apathy in 22% [3]. With increasing pressure on consultation time, unless the clinician already suspects a particular psychiatric problem, it is impractical to broadly screen patients using multiple validated rating scales. While this may be a more thorough method, a single questionnaire that can quickly screen for a range of psychiatric problems will be a useful and novel approach to guide appropriate further in-depth assessment.

We postulated that a single abbreviated questionnaire screening for common psychiatric features could adequately simulate the content of several validated rating scales when applied in mild to moderate PD. We conducted this pilot study in a population of patients with PD, aiming

- To develop the Brief Psychiatric Questionnaire (BPQ), which consists of a small number of questions screening for common psychiatric manifestations in PD
- To test content validity of the BPQ against validated rating scales
- To test repeatability of the BPQ

## 2. Material and Methods

### 2.1. Overview

This study was approved by the Alfred and Royal Melbourne Hospitals' Human Research and Ethics Committees. All eligible patients provided informed consent.

### 2.2. Subjects and Data Collection

Consecutive outpatients with idiopathic PD fulfilling the United Kingdom PD Society Brain Bank diagnostic criteria [4] from two movement disorders clinics were assessed for suitability for the study regardless of the presence of psychiatric diagnoses. Patients deemed by the clinician as unable to independently complete the questionnaires due to limited English proficiency, visual impairment, diminished hand function, severe PD (Hoehn and Yahr Stages 4-5), and dementia were excluded.

### 2.3. Brief Psychiatric Questionnaire

A literature review identified anxiety, depression, and apathy as the most common psychiatric manifestations in early stages of PD [1, 5–8]. A pool of items targeting these three problems was drafted based on key words used by patients in relation to various psychiatric complaints as identified from consultation. Items that were felt to be ambiguous and unclear were either rephrased or eliminated. These questions covered a broad range of complaints and focuses including mood, agitation, emotional control, motivation, level of interest, anxiety, self-perception, and sensation seeking. A final pool of 47 items was subjected to testing. Items broadly fell into the following categories: anxiety (5 items), apathy (9 items), depression (11 items), sensation seeking (13 items), and emotional control (9 items).

A visual analogue scale (VAS) format was chosen to allow ease of administration, particularly with a view to allow easy transference for use on mobile devices in the future. The VAS consisted of a fixed horizontal line of 10 cm with one end labeled as “Not at all” while the other end labeled as “A lot.” In response to each question, the patient was asked to place a cross on the scale to describe how they have felt in the last week. Depending on the nature of the question, scoring could be in either direction. For example, under the category of apathy, the question “Do you put a lot of effort into things?” would yield a negative score as it relates inversely to the degree of apathy. This pool of items was then reduced by statistical methods as described below, to form the BPQ.

### 2.4. Procedure

Development of the BPQ was conducted in Cohort 1, a large group of patients with PD. Clinical characteristics, levodopa equivalent daily dose (LEDD), and the Movement Disorders Society-sponsored revision of the Unified Parkinson's Disease Rating Scale motor examination (MDS-UPDRS-III) [9] were documented. In the ON state, patients were presented with the preliminary pool of 47 questions. Following this, patients completed three validated questionnaires—Geriatric Depression Scale (Short form) [10] (GDS); State Trait Anxiety Inventory (Form Y2) [11] (STAI-Y2), and Apathy Evaluation Scale [12] (AES). These three scales were deemed suitable for clinical and research use in PD patients by the Movement Disorders Society task forces [11, 13, 14]. These data were used to identify questions for inclusion in the BPQ. As the aim of this study was to determine content validity of the BPQ against the three reference scales, formal psychiatric diagnoses were not sought.

Content validity and repeatability of the BPQ were assessed in a second cohort of patients with PD. This cohort completed the BPQ as well as the three validated questionnaires outlined above. One to two weeks later, the BPQ was repeated.

### 2.5. Statistical Analysis

All statistical analysis was performed using IBM SPSS version 22 (IBM Corporation, Armonk, New York, 2013).

Factor analysis: using data from Cohort 1, principal component analysis (PCA) was applied to each “cluster” of items examining anxiety, apathy, depression, sensation seeking, and emotional control. Prior to commencing PCA, data suitability was assessed. Basic requirements for each cluster of questions were met with most interitem correlation coefficients exceeding 0.3. Kaiser-Meyer-Olkin values all exceeded the recommended value of 0.6 and Bartlett's test of sphericity reached statistical significance in each case [15]. Items forming the key components identified from PCA were retained while other items were discarded. These retained items were then correlated against the total score of each of the three reference scales. Items bearing the highest correlation were included in linear regression to generate prediction model for each reference scale. Least significant items were eliminated sequentially until only statistically significant items remained. This process further refined the question pool to form the final BPQ.

Receiver operating curves (ROC) were constructed to assess the performance of BPQ items in predicting GDS ≥ 5 [10] and the highest decile of STAI-Y2 and AES total scores. Optimal cutoff for BPQ-Depression, BPQ-Anxiety, and BPQ-Apathy items were identified based on best sensitivity and specificity of prediction.

Content validity of BPQ was tested in Cohort 2. Based on BPQ scores, predicted outcomes for each of the three validated questionnaires were calculated using linear regression models generated earlier. To measure validity, correlation between actual and predicted outcome of each of the three validated questionnaires was obtained. In addition, prediction efficiency for each group of BPQ items was determined based on calculated optimal cutoff scores. Repeatability was assessed using intraclass correlation coefficient (ICC).

## 3. Results

Cohorts 1 and 2 consisted of ninety-five and thirty-three patients, respectively. Baseline characteristics are summarized in Table 1.

### 3.1. Development of BPQ

#### 3.1.1. Factor Analysis

For each cluster of questions, PCA revealed the presence of one clear component with eigenvalue exceeding one and the scree plot showing a break after the second component. The total variance explained by the one-component solution for each group of questions was as follows: 49% for depression, 57% for anxiety, and 40% for apathy.

#### 3.1.2. Linear Regression

The items that loaded most heavily in each of the one-component solutions identified on PCA were retained. Items that correlated strongest against the final score of each of the three validated questionnaires were subjected to linear regression to generate prediction models. The final prediction models were found to explain 52% of variation of GDS (*R*^2^ = 0.521, *F*(2,94) = 49.97, *p* < 0.0001), 65% of variation of STAI-Y2 (*R*^2^ = 0.652, *F*(4,94) = 42.08, *p* < 0.0001) and 14% of variation of AES (*R*^2^ = 0.135, *F*(1,94) = 14.51, *p* < 0.0001). Following this process of data reduction, five questions were retained for further testing, forming three groups (BPQ-Anxiety, BPQ-Depression, and BPQ-Apathy) (Table 2).

#### 3.1.3. Performance of BPQ

ROCs were constructed to assess how well BPQ-Depression predicted GDS ≥ 5 and how well each corresponding BPQ group predicted the highest decile of STAI-Y2 and AES total scores. Area under the curve (AUC) for each group of BPQ items was good to excellent (0.736–0.979, *p* < 0.0001). Optimal cutoff scores for each group of BPQ items were selected and subsequently applied to Cohort 2. BPQ-Depression and BPQ-Anxiety performed particularly well, correctly identifying 88% of patients with GDS ≥ 5 and 91% of patients scoring in the highest decile on STAI-Y2. BPQ-Apathy did not perform well and had an efficiency coefficient of 0.51 (Table 3).

### 3.2. Content Validity

Content validity of the BPQ was assessed in Cohort 2. Using the generated linear regression models based on BPQ items, predicted outcomes of GDS, STAI-Y2, and AES were calculated. The correlation between predicted and actual outcomes of the three validated questionnaires was moderate to strong and highly significant (*r* = 0.464 − 0.733, *p* < 0.004) (Table 4).

### 3.3. Test-Retest Repeatability

Repeatability of BPQ items was assessed in Cohort 2. Moderate to high intraclass correlation coefficient (0.47–0.772, *p* < 0.001) was observed across repeated testing (Table 5).

Based on the above results, only BPQ-Depression and BPQ-Anxiety (Questions 1–4) were retained in the final BPQ.

## 4. Discussion

Numerous questionnaires examining psychiatric manifestations of PD are currently in use. However, a screening tool that is brief and multifaceted is lacking, and the BPQ has the potential to fill this gap. Through four simple questions, the BPQ is able to accurately identify patients at risk of depression and anxiety who scored highly on GDS and STAI-Y2 with good repeatability. Results exceeding the calculated scoring thresholds of the relevant BPQ groups should alert the clinician to further test for emerging depression and anxiety.

PD poses a unique dilemma as many psychiatric problems overlap with its physical manifestations (e.g., fatigue, psychomotor slowing, sleep disturbances, and loss of appetite) and sequelae (e.g., social withdrawal, hopelessness, and helplessness) [11, 13, 14]. In addition, nonmotor fluctuations are well reported with emergence of anxiety and low mood in OFF periods [16]. Therefore, the clinician must first exclude PD related symptoms in order to identify patients who may have true psychiatric problems. The gold standard of psychiatric diagnosis is structured interview using the Diagnostic and Statistical Manual of Mental Disorders (DSM-5) or other established diagnostic criteria. As this is a time and resource consuming process, patients must first be selected by using screening scales or clinical assessment. A plethora of validated scales and questionnaires examining various psychiatric problems exist, and enormous efforts have been devoted to identify ones that may be useful in PD for the purposes of screening and measuring symptom severity [11, 13, 14].

Most established rating scales are generally unidimensional, and many are too lengthy for screening purpose. The Neuropsychiatric Inventory [17] is one instrument that measures multiple behavioural and psychiatric domains. However, it is intended for patients with dementia, and its clinimetric properties in patients with PD are unknown [18]. Another example is the MDS-UPDRS Part 1A, which comprises six items assessing the nonmotor aspects of PD, specifically cognitive impairment, hallucination, depression, anxiety, apathy, and dopamine dysregulation [9]. However, completion of this part of the scale requires a rater to perform a semistructured interview, estimated to take ten minutes [9]. While there are abbreviated self-reporting scales such as the Generalized Anxiety Disorder scale (GAD-7) [19] and Patient Health Questionnaire (PHQ-2 and PHQ-9) [20], they were developed for use in primary care and have not been validated in PD patients. Furthermore, a number of questions focus on physical symptoms (e.g., feeling of restlessness, poor appetite, and feeling tired) that overlap substantially with symptoms of PD. Therefore, a concise single index measuring multiple constructs can broaden and simplify the screening process in patients with PD.

The BPQ is self-administrated and takes only one or two minutes to complete. A self-reporting, VAS format was chosen to maximize ease and minimize time of administration. We felt that VAS is more appropriate to measure symptoms that exist as a continuum rather than categories. Furthermore, VAS format can be more sensitive to change in an individual [21]. To reduce the number of included items, it was important to recognize that symptoms of different conditions may overlap, especially those of anxiety, depression, and apathy. Consistent with this clinical observation, items 1 and 2 were found to measure both depression and anxiety. Therefore, rather than prespecifying items to measure a particular problem, we took advantage that some items may have multiple dimensions. This allows the number of items in the questionnaire, hence time to complete the questionnaire, to be minimized.

While the BPQ was able to identify patients at risk of anxiety and depression, attempts to recognize apathetic patients proved much more challenging. Symptoms of anxiety, depression, and apathy are often intertwined and difficult to distinguish, even though apathy can occur in the absence of depression in patients with PD [14]. Apathy is difficult to characterize, and the concept of a disorder of motivation is equally difficult to operationalize [22]. These reasons could account for the inability of the BPQ to identify patients scoring highly on the AES. When compared to Cohort 1, patients in Cohort 2 were older with more motor impairment despite having shorter disease duration. Despite the differences in disease characteristics, BPQ-Anxiety and BPQ-Depression still performed well, reinforcing the feasibility of broader application of the BPQ in PD patients.

A number of limitations should be highlighted. We have chosen three reference scales [11, 13, 14] that we felt would have served a similar screening purpose as the BPQ. It is important to emphasize that the main aim of this study was to assess how well the BPQ reflects the content of three full-length reference scales and not to assess the actual diagnostic accuracy of the BPQ. However, further testing of criterion validity against the “gold standard” of structured interview and formal psychiatric diagnoses will no doubt further strengthen the clinimetrics of the BPQ in patients with PD. Our sample size is small for the application of principal component analysis. However, it has been suggested that a smaller sample size should be sufficient if solutions have several high loading marker variables [23], which was the case in this study. Further evaluation in larger patient population will enhance the generalizability of BPQ. ON and OFF states were not controlled for during repeatability testing, and this may have contributed to greater variability of responses, particularly in patients with nonmotor fluctuations.

## 5. Conclusion

The BPQ differs from other established scales in its brevity and multifaceted nature. Despite its abbreviated format, the BPQ was able to identify patients at risk of anxiety and depression with high accuracy. Practically, existing unidimensional questionnaires are more suitable to be used when there is already a degree of suspicion of a particular psychiatric problem. On the other hand, the BPQ can be broadly and indiscriminately applied to all patients. It is not designed to be a diagnostic tool or a replacement of more detailed psychiatric assessment but rather to give the clinician a signal of emerging depression and anxiety. As such, we see the BPQ as a preconsultation tool that can be reliably administered on repeated occasions to prompt further psychiatric assessment.

## Figures and Tables

**Table 1:** Baseline characteristics of cohorts 1 and 2.

	Cohort 1 (*n*=95) (mean, range, SD)	Cohort 2 (*n*=33) (mean, range, SD)	*p* value
Age (years)	65 (38–87, 9.3)	74 (49–89, 10.2)	<0.0001
Male (%)	48 (51%)	22 (66%)	0.132
Disease duration (years)	8.6 (2–24, 4.8)	4 (1–12, 3)	<0.0001
LEDD (mg)	920 (150–3064, 544)	600 (150–1550, 325)	0.002
ON-state MDS-UPDRS-III	20 (1–47, 9.5)	35 (15–66, 11)	<0.0001

**Table 2:** (A) questions retained for further testing and (B) combination of questions used in the prediction models for each validated questionnaire.

(A) Final questions considered for the Brief Psychiatric Questionnaire
(1) Do you often feel withdrawn?
(2) Do you often feel that your life is empty?
(3) Do you worry excessively over little things?
(4) Do you put a lot of effort into things?
(5) Do you usually start looking for something thrilling or exciting when nothing new is happening?

(B) Prediction models
GDS—questions 1 and 2 (BPQ-Depression)
STAI-Y2—questions 1, 2, 3 and 4 (BPQ-Anxiety)
Apathy scale—question 5 (BPQ-Apathy)

**Table 3:** Performance of BPQ groups assessed by area under the curve (AUC) and sensitivity, specificity, and efficiency coefficient of optimal cutoff scores.

Targets	AUC	Optimal cutoff scores	Sensitivity (%)	Specificity (%)	Efficiency coefficient
GDS ≥ 5	0.896 (<0.0001)	BPQ-Depression > 6	75	90	0.88
Highest decile of STAI-Y2	0.979 (<0.0001)	BPQ-Anxiety > 17	100	93	0.91
Highest decile of apathy scale	0.736 (0.015)	BPQ-Apathy > 7	70	65	0.51

**Table 4:** Correlation of predicted and actual scores for each validated questionnaire.

	Correlation of predicted and actual scores	*p* value
GDS	0.733	<0.0001
STAI-Y2	0.493	0.004
Apathy scale	0.464	0.004

**Table 5:** Intraclass correlation coefficient of the five questions considered for the BPQ.

BPQ items	ICC (95% confidence interval, *p*)
Question 1	0.648 (0.355–0.825, <0.0001)
Question 2	0.772 (0.555–0.891, <0.0001)
Question 3	0.598 (0.281–0.797, 0.0005)
Question 4	0.47 (0.109–0.722, 0.007)
Question 5	0.556 (0.222–0.773, 0.01)

## Data Availability

The data used to support the findings of this study are available from the corresponding author upon request.

## References

[1] Stankovic I., Stefanova E., Tomic A. (2016). Psychiatric symptoms in the initial motor stage of Parkinson’s disease. *Journal of Neuropsychiatry and Clinical Neurosciences*.

[2] Brown R. G., Landau S., Hindle J. V. (2011). Depression and anxiety related subtypes in Parkinson’s disease. *Journal of Neurology, Neurosurgery & Psychiatry*.

[3] Kirsch-Darrow L., Fernandez H. F., Marsiske M., Okun M. S., Bowers D. (2006). Dissociating apathy and depression in Parkinson disease. *Neurology*.

[4] Gibb W. R., Lees A. J. (1988). The relevance of the lewy body to the pathogenesis of idiopathic Parkinson’s disease. *Journal of Neurology, Neurosurgery & Psychiatry*.

[5] Antonini A., Siri C., Santangelo G. (2011). Impulsivity and compulsivity in drug-naïve patients with Parkinson’s disease. *Movement Disorders*.

[6] Ravina B., Camicioli R., Como P. G. (2007). The impact of depressive symptoms in early Parkinson disease. *Neurology*.

[7] Pedersen K. F., Alves G., Brønnick K., Aarsland D., Tysnes O.-B., Larsen J. P. (2010). Apathy in drug-naïve patients with incident Parkinson’s disease: the Norwegian ParkWest study. *Journal of Neurology*.

[8] Weintraub D., Simuni T., Caspell-Garcia C. (2015). Cognitive performance and neuropsychiatric symptoms in early, untreated Parkinson’s disease. *Movement Disorders*.

[9] Goetz C. G., Fahn S., Martinez-Martin P. (2007). Movement disorder society-sponsored revision of the unified Parkinson’s disease rating scale (MDS-UPDRS): process, format, and clinimetric testing plan. *Movement Disorders*.

[10] Yesavage J. A. (1988). Geriatric depression scale. *Psychopharmacology Bulletin*.

[11] Leentjens A. F. G., Dujardin K., Marsh L. (2008). Anxiety rating scales in Parkinson’s disease: critique and recommendations. *Movement Disorders*.

[12] Marin R. S., Biedrzycki R. C., Firinciogullari S. (1991). Reliability and validity of the apathy evaluation scale. *Psychiatry Research*.

[13] Schrag A., Barone P., Brown R. G. (2007). Depression rating scales in Parkinson’s disease: critique and recommendations. *Movement Disorders*.

[14] Leentjens A. F. G., Dujardin K., Marsh L. (2008). Apathy and anhedonia rating scales in Parkinson’s disease: critique and recommendations. *Movement Disorders*.

[15] Kaiser H. F. (1974). An index of factorial simplicity. *Psychometricka*.

[16] Raudino F. (2001). Non motor off in Parkinson’s disease. *Acta Neurologica Scandinavica*.

[17] Cummings J. L., Mega M., Gray K., Rosenberg-Thompson S., Carusi D. A., Gornbein J. (1994). The neuropsychiatric inventory: comprehensive assessment of psychopathology in dementia. *Neurology*.

[18] Fernandez H. H., Aarsland D., Fénelon G. (2008). Scales to assess psychosis in Parkinson’s disease: critique and recommendations. *Movement Disorders*.

[19] Spitzer R. L., Kroenke K., Williams J. B. W., Löwe B. (2006). A brief measure for assessing generalized anxiety disorder. *Archives of Internal Medicine*.

[20] Spitzer R. L., Kroenke K., Williams J. B. (1999). Validation and utility of a self-report version of PRIME-MD: the PHQ primary care study. Primary care evaluation of mental disorders. Patient health questionnaire. *JAMA*.

[21] Grant S., Aitchison T., Henderson E. (1999). A comparison of the reproducibility and the sensitivity to change of visual analogue scales, Borg scales, and Likert scales in normal subjects during submaximal exercise. *Chest*.

[22] Merrilees J., Dowling G. A., Hubbard E., Mastick J., Ketelle R., Miller B. L. (2013). Characterization of apathy in persons with frontotemporal dementia and the impact on family caregivers. *Alzheimer Disease & Associated Disorders*.

[23] Tabachnick B. G. F. L. (2013). *Using Multivariate Statistics*.

